# Data on the proliferation and differentiation of C2C12 myoblast treated with branched-chain ketoacid dehydrogenase kinase inhibitor

**DOI:** 10.1016/j.dib.2020.105766

**Published:** 2020-05-26

**Authors:** Yoriko Sato, Hayato Tate, Fumiaki Yoshizawa, Yusuke Sato

**Affiliations:** a Center for Bioscience Research and Education, Utsunomiya University, Tochigi, Japan; b Department of Agrobiology and Bioresources, Utsunomiya University, Tochigi, Japan

**Keywords:** C2C12 myoblast, Proliferation, Differentiation, Branched chain amino acids, Branched chain ketoacid dehydrogenase kinase inhibitor

## Abstract

The catabolism of branched chain amino acids (BCAAs) is mainly carried out in skeletal muscle myofibers. It is mediated by branched chain aminotransferase 2 and branched chain alpha ketoacid dehydrogenase (BCKDH) in mitochondria for energy supply, especially during exercise. BCKDH kinase (BCKDK) is a negative regulator of BCAAs catabolism by its inhibitory phosphorylation of the BCKDH E1a subunit. The data presented in this article are related to the research article that we previously have reported entitled “Energy metabolism profile of the effects of amino acid treatment on skeletal muscle cells: Leucine inhibits glycolysis of myotubes” (Suzuki et al., 2020)[1]. In this report, we have demonstrated that 1hour treatment of BT2, an inhibitor of BCKDK, decreased the glycolysis of C2C12 differentiated myotubes compared to the control. Although BCAAs metabolism is basically assumed to be carried out in differentiated myofibers, BCKDK is expressed in both undifferentiated myoblasts and differentiated myotubes, and the biological and physiological significance of BCAAs metabolism in myoblasts is still unclear. Present data demonstrate an in vitro assessment of BT2 on C2C12 myoblasts proliferation and differentiation. The data suggest that activation of BCAAs catabolism by the BCKDK inhibitor BT2 impairs C2C12 myoblasts proliferation and differentiation.

Specifications table

**Table:** 

Subject	Cell Biology
Specific subject area	Muscle cell biology, Branched chain amino acids metabolism
Type of data	Graph Figure
How data were acquired	Immunoblot, qRT-PCR, Microscopy, Cell proliferation assay
Data format	Analyzed
Parameters for data collection	C2C12 myoblasts were treated with BT2 to activate branched chain amino acids metabolism both during proliferation and differentiation
Description of data collection	C2C12 myoblasts were treated with BT2 (0-100 μM). The effect of BT2 on cell proliferation was evaluated by Cell Counting Kit-8 (CCK-8). The effect of BT2 on myogenic differentiation was evaluated by measuring the differentiation markers using immunoblot and qRT-PCR.
Data source location	Utsunomiya university Tochigi Japan
Data accessibility	Data are presented with this article
Related research article	Reiko Suzuki, Yoriko Sato, Kodwo Amuzuah Obeng, Daisuke Suzuki, Yusuke Komiya, Shin-ichi Adachi, Fumiaki Yoshizawa, Yusuke Sato Energy metabolism profile of the effects of amino acid treatment on skeletal muscle cells: Leucine inhibits glycolysis of myotubes. Nutrition, doi.org/10.1016/j.nut.2020.110794

## Value of the data

• The data provide the possibility that activation of branched chain amino acids catabolism by the BCKDK inhibitor BT2 may suppress C2C12 myoblasts proliferation and differentiation.
• Since BCAAs metabolism is basically assumed to be carried out in differentiated myofibers, the biological and physiological significance of BCAAs metabolism in myoblasts is still unclear.
• The data is valuable for researchers interested in the relationship between branched chain amino acids metabolism and physiology of skeletal muscle cell.
• The data help researchers design experiments examining C2C12 myoblasts proliferation and differentiation in response to drug.

## 1 Data Description

**Fig. 1:**
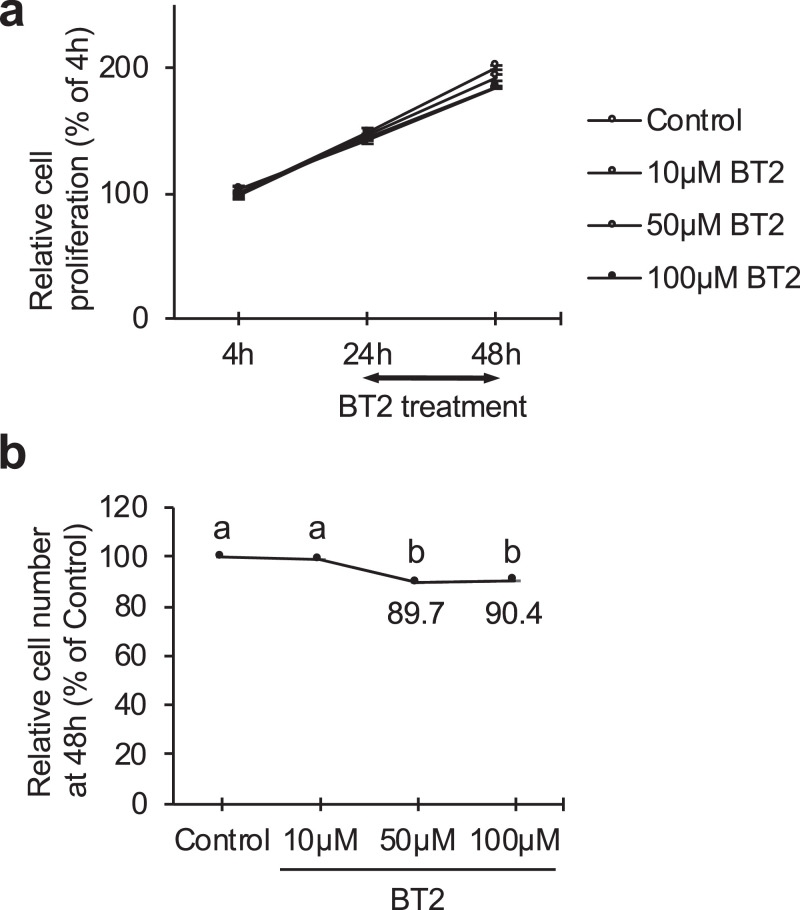
The effect of BT2 on C2C12 myoblast proliferation. C2C12 myoblasts were cultured in DMEM containing 1% FBS and BT2 (10, 50, 100 μM). Cell Counting Kit-8 was used to measure the relative number of myoblasts. a) Relative cell proliferation rates were presented as percentage of 4h. b) Relative cell number 24 h after the treatment of BT2 was presented. Different superscripts indicate a significant difference between 2 groups. All assessments of significance were performed with 1-way ANOVA with Tukey post hoc test (p < 0.05). Values are means ± SEM (n = 12–14).

**Fig. 2:**
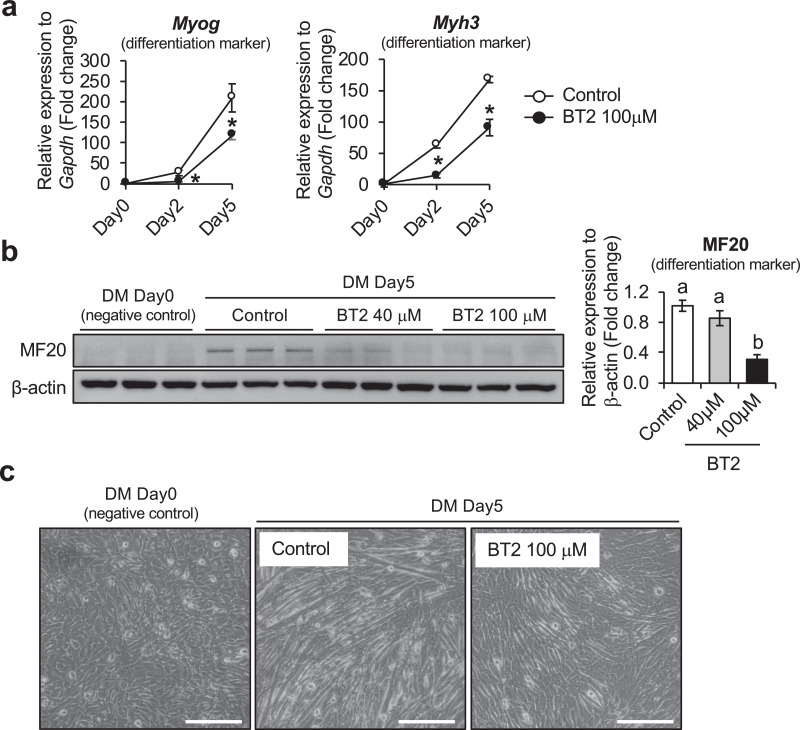
The effect of BT2 on C2C12 myoblast differentiation. a) Time-course qRT-PCR analysis of the effect of BT2 treatment (100 μM) on the expression of myogenic differentiation markers (*Myh3* and *Myog*) of C2C12 myoblasts. mRNA expression levels were calculated relative to *Gapdh* and the data are expressed as a fold-increase. Significance was determined with the two-tailed Student's t-test (vs. control, *p < 0.05) (n = 6). Values are expressed as means ± SEM. b) The effect of BT2 treatment (40 μM and 100 μM) on total MyHC expression (anti-MF20) of C2C12 myoblasts for 5 days after induction of differentiation. Myoblasts at DM 0day (cultured in growth media) is used as negative control. Graph shows the relative intensity of each band after normalization to β-actin. Different superscripts indicate a significant difference between 2 groups. All assessments of significance were performed with 1-way ANOVA with Tukey post hoc test (p < 0.05) (n = 3). Values are expressed as means ± SEM. c) Representative images of Control and BT2-treated (100 μM) C2C12 myoblasts for 5 days after induction of differentiation. Myoblasts at DM day0 was shown as negative control. Bar = 100 μm.

Recently we have reported that activation of branched chain amino acids catabolism by BT2, a BCKDK (branched chain ketoacid dehydrogenase kinase) inhibitor, impaired the glycolysis of C2C12 myotubes [1,2]. BCKDK is expressed in both C2C12 myoblasts and differentiated myotubes. Here, we present data regarding the effect of BT2 on C2C12 myoblast proliferation and myogenic differentiation. The data in Fig. 1 show the comparison of cell proliferation rate between control and BT2-treated myoblasts. Data in Fig. 1 were obtained by Cell Counting Kit-8. The data in Fig. 2 show the comparison of myogenic differentiation between control and BT2-treated myoblasts after induction of differentiation by reducing the serum concentration. Data in Fig. 2 were obtained by immunoblot, qRT-PCR and microscopy.

## 2 Experimental Design, Materials, and Methods

To investigate the effect of BCAAs catabolism on myoblasts proliferation and myogenic differentiation, C2C12 myoblasts were treated with BT2, an inhibitor for BCKDK. For the evaluation of myoblasts proliferation, myoblasts were cultured for 24 hours and then relative cell proliferation rate was measured by Cell Counting Kit-8. For the evaluation of myogenic differentiation, myoblasts were collected at day 0, 2 and 5 after induction of differentiation and then myogenic marker genes and protein expression were measured by qRT-PCR and immunoblot.

### 2.1 Cell culture and reagents

C2C12 myoblasts were purchased from ATCC (Manassas, VA, USA). C2C12 myoblasts at early passage (3-10) were used for experiment. Cells were maintained in DMEM supplemented with 10% fetal bovine serum, 1% penicillin/streptomycin mixture at 37 °C with 5% CO_2_. For myogenic differentiation, myoblasts were cultured in 2% HS-DMEM until myotubes formed (5 days) after the cells reached 80-90% confluency. For gene and protein expression analyses, cells were seeded on 12-well miniplates (n = 6, each group) or 6-well miniplates (n = 3, each group), respectively. BT2 (3,6-dichlorobenzo[b]thiophene-2-carboxylic acid) (Axon Medchem, Groningen, Netherland) was used to inhibit BCKDC kinase for the activation of BCAAs catabolism [2].

### 2.2 Cell proliferation assay

Cell proliferation assay was assessed with a Cell Counting Kit-8 assay (Dojindo Laboratories, Kumamoto, Japan) according to the manufacture's protocol with slight modifications [3]. C2C12 myoblasts were seeded in 96-well miniplates at a density of 3000 cells/well in DMEM containing 10% FBS for 24 hours. The culture medium was removed and replaced with DMEM containing 1% FBS and BT2 (10-100 μM). After 24 hours of culture, cell proliferation was assessed using Cell Counting Kit-8.

### 2.3 RNA extraction and quantitative real-time polymerase chain reaction

Expression of target and reference genes was measured using a quantitative real-time polymerase chain reaction (qRT-PCR) according to the previous report [4]. *Gapdh* was used as the reference gene. The significance of differences in mRNA was calculated by 2-∆∆Ct method. Total RNAs were isolated from 6 individual wells of cultured C2C12 myoblasts according to the regular Trizol-chloroform protocol. cDNA was synthesized from 1 μg of total RNA by a reverse-transcriptase iScript (Bio-Rad, Hercules, CA, USA), and qRT-PCR was performed using LightCycler 96 (Roche Diagnostics, Mannheim, Germany). The primer sets were designed by Primer3. The primer sequences are as follows: Gapdh forward, TTGCCATCAACGACCCCTTC; Gapdh reverse, TTGTCATGGATGACCTTGGC; *Myog* forward, ACCTTCCTGTCCACCTTCAG; *Myog* reverse, CACCGACACAGACTTCCTCT; *Myh3* forward, CAATAAACTGCGGGCAAAGAC; *Myh3* reverse, CTTGCTCACTCCTCGCTTTCA.

### 2.4 Protein extraction and immunoblot analyses

Proteins were extracted from 3 individual wells of cultured C2C12 myoblasts of each group. The samples were homogenized in SDS sample buffer containing 125 mm Tris–HCl pH 6.8, 5% β-mercaptoethanol, 2% SDS and 10% glycerol. Extracted proteins were separated on acrylamide gels, and then transferred onto PVDF membranes (GE Healthcare). A blocking solution of 5% BSA was used. The chemidoc XRS Imager (Bio-rad) was used for evaluating the detected bands. Total myosin heavy chain was measured by MF20 antibody (eBioscience, 14–6503–82, dilution 1:1000) to determine the differentiation level of C2C12 myoblasts. β-actin was used as internal standard (Cell Signaling Technology, #4967, dilution 1:1000).

### 2.5 Statistical analysis

All data are presented as means ± SEM. P values less than 0.05 were considered significant and all assessment of significance was performed with unpaired 2-tailed Student's t-test or 1-way analysis of variance (ANOVA) with Tukey post hoc test using Prism6 (GraphPad Software, La Jolla, CA, USA).

## Declaration of Competing Interest

The authors declare that they have no known competing financial interests or personal relationships which have, or could be perceived to have, influenced the work reported in this article.

## Appendix Supplementary materials

Image, application 1
